# Identifying specific social challenges of rare diseases: current challenges and issues

**DOI:** 10.1186/1750-1172-9-S1-O29

**Published:** 2014-11-11

**Authors:** Dorica Dan, Raquel Castro

**Affiliations:** 1 Romanian National Alliance for Rare Diseases, Zalau, Romania; 2 Romanian Prader-Willi Association, Zalau, Romania; 3 European Organisation for Rare Diseases – EURORDIS, Paris, France; 4 European Commission Expert Group on Rare Diseases – DG Health and Consumers, Brussels, Belgium

## Background

EURORDIS Care Survey program conducted with over 12000 patients in 23 countries (2002-2008) has concluded that «social security systems are usually designed around common diseases and are not flexible enough to take into consideration unprecedented health needs» [1] and has provided a few insights into rare diseases (RD) patients and families social challenges. Further data collection and literature review is needed in order to assess more accurately these social challenges.

## Objective

To compile information on social challenges of RD patients and their families.

## Method

The identification of challenges is performed based on a literature review of:

- Communication from the Commission on Rare Diseases: Europe's Challenges (2008) [2];

- Council Recommendation on an Action in the Field of Rare Diseases (2009) [3];

- Communication from the Commission: European Disability Strategy 2010-2020 (2010) [4];

- EUROPLAN Report on 15 National Conferences (2010-2011) [5];

- EURORDISCare Survey Programme (2002-2008);

- ‘Rare Diseases: Addressing the Need for Specialised Social Services and Social Policies’ [6].

## Results

Main social challenges identified:

- Lack of long term, funded and sustainable policies and structures at national level for the integration of patients with RD into social services and policies;

- Weak coordination between health and psychosocial complementary care, between central and regional/local infrastructures, leading to a consequent lack of multidisciplinary holistic approach;

- Lack of systems to accurately evaluate patients’ disability degree and consequent lack of adequate compensation measures;

- Lack of information and understanding of patients’ disabilities and corresponding implications in patients’/families’ daily lives;

- Lack of training of social sector professionals to deal with rare, complex cases, resulting in unprepared services/structures and insufficient sharing of best practices;

- Scarcity of social services and social policies/benefits. Difficulties in accessing services;

- Lack of case managers guiding patients/families to access the different types of care and structures;

- Lack of personalised/flexible measures and policies;

- Lack of measures to remove burden from family in daily care;

- Lack of systems to deal with transition from adulthood to childhood and ageing.

## Conclusion

To improve the access from RD patients/families to adequate and high quality social policies and services there is a need to address these current social challenges by shaping national policies and implementing solutions at MS level. Guiding principles to address some of these challenges are currently being compiled within the European Committee of Experts on Rare Diseases Join Action Work Package 6.
